# 细胞因子与肿瘤标志物联合检测对孤立性肺结节良恶性鉴别诊断的价值

**DOI:** 10.3779/j.issn.1009-3419.2021.102.20

**Published:** 2021-06-20

**Authors:** 婕 石, 璇 刘, 宗娟 明, 维 李, 欣 吕, 侠 杨, 煜 王, 梦颖 张, 拴盈 杨

**Affiliations:** 710061 西安，西安交通大学第二附属医院呼吸与危重症医学科 Department of Pulmonary and Critical Care Medicine, The Second Affiliated Hospital of Xi'an Jiaotong University, Xi'an 710061, China

**Keywords:** 孤立性肺结节, 肿瘤标志物, 细胞因子, 联合检测, 诊断, Solitary pulmonary nodule, Tumor markers, Cytokines, Combined detection, Diagnosis

## Abstract

**背景与目的:**

近年来，孤立性肺结节（solitary pulmonary nodule, SPN）受到越来越多的关注，部分肺结节被认为是早期肺癌，但如何鉴别肺结节良恶性却是亟待解决的临床难题。本研究旨在探讨细胞因子与肿瘤标志物联合检测对SPN良恶性的鉴别诊断价值，从而提高SPN诊断的准确性。

**方法:**

纳入81例诊断明确的SPN患者作为研究对象，收集病例的一般临床资料、结节影像学特征、病理学诊断资料、细胞因子系列和肿瘤标志物表达水平。利用单因素和多因素分析筛选可预测肺结节性质的影响指标，并用二元*Logistic*回归分析构造联合指标；绘制受试者工作特征曲线（receiver operating characteristic curve, ROC），计算曲线下面积及相应的灵敏度、特异度、阳性预测值、阴性预测值和准确率。

**结果:**

一般临床资料分析示恶性结节出现在右肺上叶的比例最高（40.4%）。恶性结节组中的癌胚抗原（carcinoembryonic antigen, CEA）、细胞角蛋白19片段（cytokeratin 19 fragment 21-1, CYFRA21-1）、白介素6（interleukin-6, IL-6）和白介素8（interleukin-8, IL-8）血清水平高于良性结节组。*Logistic*回归分析提示，CEA、IL-6、IL-8为预测恶性结节的独立危险因子。ROC曲线分析表明，单项指标CEA、IL-6和IL-8的曲线下面积分别为0.642、0.684和0.749，CEA+IL-6+IL-8联合检测曲线下面积更大，检测效能更高。

**结论:**

CEA、IL-6和IL-8为恶性结节的独立危险因素。细胞因子和肿瘤标志物联合检测在SPN良恶性鉴别诊断中具有一定的价值。其中CEA+IL-6+IL-8联合检测的诊断价值最高。

## Abstract (en)

**Background and objective:**

Solitary pulmonary nodule has received increasing attention in recent years. A couple of lung nodules have been recognized as primary malignant tumors, which leads to an urgent need in enhancing the diagnosis of benign/malignant lung nodules at clinical settings. This study aims to explore the value of the combined detection of cytokines and tumor markers in differencing benign and malignant solitary pulmonary nodules in diagnose.

**Methods:**

With 81 solitary pulmonary nodules cases with a clear diagnosis, the general clinical data, nodule imaging features, pathological diagnosis data, serological index cytokine series and tumor marker expression levels were collected in groups. Both single factor and multi-factors analysis were conducted to screen out the serum influence indexes that can predict the malignant probability of lung nodules, and mean while binary logistic regression analysis was used to construct joint indexes; After receiver operating characteristic curve (ROC) was drawn, the area under the curve and the corresponding sensitivity, specificity and positive of each index predicted value, negative predicted value and accuracy could be calculated with a view to determine the statistical significance of area under the curve (AUC).

**Results:**

There are differences in the distribution of malignant solitary pulmonary nodules at different locations, with the highest proportion of the right upper lobe (40.4%). The serum levels of carcinoembryonic antigen (CEA), cytokeratin 19 fragment 21-1 (CYFRA21-1), interleukin-6 (IL-6), interleukin-8 (IL-8) in the malignant nodule group were higher than those in the benign nodule group. *Logistic* regression analysis suggests that CEA, IL-6 and IL-8 are independent risk factors for predicting malignant nodules. ROC curve analysis shows that the areas under the curve of the individual indicators CEA, IL-6 and IL-8 are 0.642, 0.684 and 0.749. The comparison result of the test efficiency of the area under the curve suggests that CEA+IL-6+IL-8 has a larger area under the curve and higher detection efficiency.

**Conclusion:**

CEA, IL-6 and IL-8 are independent risk factors for malignant solitary pulmonary nodules. The combined detection of cytokines and tumor markers has played a role in the differential diagnosis of benign and malignant lung nodules. The diagnostic value of the combined detection of CEA+IL-6+IL-8 is the highest.

肺癌是世界范围内最常见的恶性肿瘤，也是癌症相关性死亡的主要原因^[1]^。根据中国国家癌症登记中心数据^[2]^显示，肺癌在男性中的发病率居于首位，在女性中的发病率仅次于乳腺癌。肺癌是癌症死亡的首要原因。早期肺癌的预后显著优于晚期肺癌，因此，对早期识别肺癌尤为重要。孤立性肺结节（solitary pulmonary nodule, SPN）是指影像学上发现的肺内直径≤3 cm的单个、局灶性、类圆形、密度增高的肺部阴影，周围被肺实质包绕，无肺不张、淋巴结增大或胸腔积液等表现^[3]^。虽然部分肺结节是良性的，但有相当一部分是早期的、潜在可治愈的恶性肿瘤^[4]^。因此，尽早识别良恶性SPN是目前临床工作的的热点和难点。

血清肿瘤标志物为肿瘤的诊断、治疗疗效及预后提供参考证据，但是，其在早期肺癌患者中敏感性较低^[5]^。为解决肿瘤标志物在早期肺癌诊断中的局限性，与其他血清生物标志物进行联合检验成为目前研究的热点。

炎性细胞因子是由多种免疫细胞和肿瘤细胞分泌的一类具有广泛生物学活性的低分子量可溶性蛋白质，可以反映肿瘤微环境和机体的免疫功能^[6]^。既往研究表明炎性因子与肿瘤密切相关，我们拟探讨细胞因子与肿瘤标志物联合检测对SPN良恶性的鉴别诊断价值，寻找可预测恶性SPN的血清生物标志物，从而提高早期肺癌诊断的准确性。

## 1 材料与方法

### 1.1 研究对象及分组

选取2019年3月-2020年1月就诊于西安交通大学第二附属医院经胸部计算机断层扫描（computed tomography, CT）发现SPN并最终明确诊断且病例资料记录完整的患者。排除标准：既往诊断为肺癌并接受治疗的患者；肺部有急性感染或者活动性肺结核病变的患者；有自身免疫性疾病，服用激素治疗的患者；有严重的心、肝、肾功能异常的患者；有其他系统恶性肿瘤史的患者。最终入选81例，依据病理结果分为良性结节组和恶性结节组，其中恶性结节组47例，肺腺癌39例，鳞癌8例；良性结节组34例。收集患者的临床资料、影像学资料、病理资料和血清学指标（包括常见肺肿瘤标志物和细胞因子系列）。

### 1.2 统计学方法

采用SPSS 18.0统计学软件、MedCalc软件和GraphPad Prism 8.0软件进行数据分析和绘图。计数资料用频数（百分比）表示，组间比较采用卡方检验或*Fisher*精确检验；符合正态分布的计量资料两组间比较采用独立样本*t*检验，多组间比较采用单因素方差分析；偏态分布的资料两组间比较采用两个独立样本的*Mann-Whitney*非参数检验；采用单因素和多因素分析筛选出预测肺结节恶性概率的独立血清指标，并用二元*Logistic*回归分析构造联合指标；绘制受试者工作特征曲线（receiver operating characteristic curve, ROC），金标准为病理结果，计算曲线下面积（area under the curve, AUC）。*P* < 0.05表明差异具有统计学意义。

## 2 结果

### 2.1 良性结节组和恶性结节组的一般临床资料分析

如表 1所示，良性结节组和恶性结节组在分布中存在显著差异（*P*=0.041），其中恶性结节在右肺上叶的比例最高（40.4%）；良性结节组和恶性结节组在性别、年龄、吸烟史、肿瘤家族史、不同直径和不同密度结节中的分布均无统计学差异（*P* > 0.05）。

**表 1:** 良性结节组和恶性结节组的一般临床资料分析 General clinical data of patients with benign or malignant solitary pulmonary nodules

Index	Benign solitary pulmonary nodules (*n*=34)	Mmalignant solitary pulmonary nodules (*n*=47)	*P*
Gender			0.668
Male	19 (55.9%)	24 (51.1%)	
Female	15 (44.1%)	23 (48.9%)	
Age (yr)			0.782
< 65	22 (64.7%)	18 (38.3%)	
≥65	12 (35.3%)	29 (61.7%)	
Smoking history			0.442
Yes	21 (61.8%)	25 (53.2%)	
No	13 (38.2%)	22 (46.8%)	
Family history of tumor			0.764
Yes	7 (20.6%)	11 (23.4%)	
No	27 (79.4%)	36 (76.6%)	
Nodules size			0.094
≤10 mm	7 (20.6%)	4 (8.5%)	
11 mm-19 mm	17 (50.0%)	19 (40.4%)	
20 mm-30 mm	10 (29.4%)	24 (51.1%)	
Nodules density			0.288
Ground glass nodules	9 (26.5%)	6 (12.8%)	
Partial solid nodules	6 (17.6%)	9 (19.1%)	
Solid nodules	19 (55.9%)	32 (68.1%)	
Location			0.041
Superior lobe of right lung	6 (17.6%)	19 (40.4%)	
Middle lobe of right lung	3 (8.8%)	3 (6.4%)	
Inferior lobe of right lung	9 (26.5%)	12 (25.5%)	
Superior lobe of left lung	5 (14.7%)	9 (19.2%)	
Inferior lobe of left lung	11 (32.4%)	4 (8.5%)	

### 2.2 细胞因子在良性结节组和恶性结节组中的表达水平

如图 1及表 2所示，白介素6（interleukin-6, IL-6）、白介素8（interleukin-8, IL-8）在恶性结节患者组中的表达水平高于良性结节组，具有统计学差异（*P* < 0.01）；IL-8在恶性结节患者组中的表达水平高于良性结节组，具有统计学差异（*P* < 0.01）；而白介素2受体（interleukin-2 receptor, IL-2R）、肿瘤坏死因子α（tumor necrosis factor-α, TNF-α）在良性结节组和恶性结节组间未见显著差异。

**图 1:**
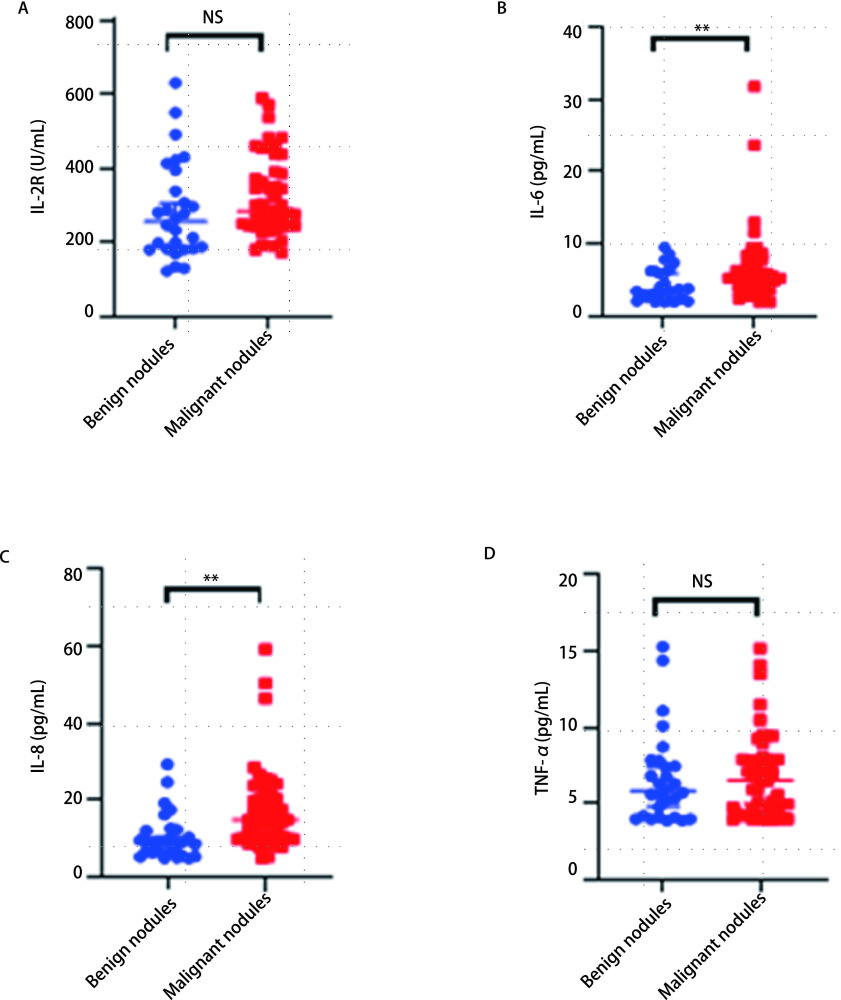
良性结节组和恶性结节组中细胞因子表达水平的比较 Comparison of cytokines expression levels of patients with benign or malignant solitary pulmonary nodules. **P < 0.01; NS: non-significant difference.

**表 2:** 细胞因子在良性结节组和恶性结节组中的表达水平 Cytokines expression level of patients with benign or malignant solitary pulmonary nodules

Item	IL-2R (U/mL)	IL-6 (pg/mL)	IL-8 (pg/mL)	TNF-α (pg/mL)
Benign solitary pulmonary nodules (*n*=34)	272.50 (183.00, 394.00)	3.45 (2.35, 6.06)	9.25 (6.57, 12.60)	5.85 (4.20, 7.40)
Malignant solitary pulmonary nodules (*n*=47)	284.00 (245.00, 391.00)	5.25 (3.57, 7.85)	14.90 (10.10, 20.30)	6.53 (4.35, 7.93)
*Z*(*t*)	-1.704	-2.651	-3.115	-0.910
*P*	0.088	0.008	0.002	0.363
IL-2R: interleukin-2 receptor; IL-6: interleukin-6; IL-8: interleukin-8; TNF-α: tumor necrosis factor-α.				

### 2.3 肿瘤标志物在良性结节组和恶性结节组中的表达水平

如图 2及表 3所示，癌胚抗原（carcinoembryonic antigen, CEA）和细胞角蛋白19片段（cytokeratin 19 fragment 21-1, CYFRA21-1）在恶性结节患者组中的表达水平高于良性结节组，具有统计学差异（*P* < 0.05）；神经元特异性烯醇化酶（neuron-specific enolase, NSE）、糖类抗原125（carbohydrate antigen 125, CA125）、胃泌素释放前肽（progastrin releasing peptide, PROGRP）、鳞癌抗原（squamous cell carcinoma antigen, SCC-Ag）在良性结节组和恶性结节组间的表达水平均无显著差异。

**图 2:**
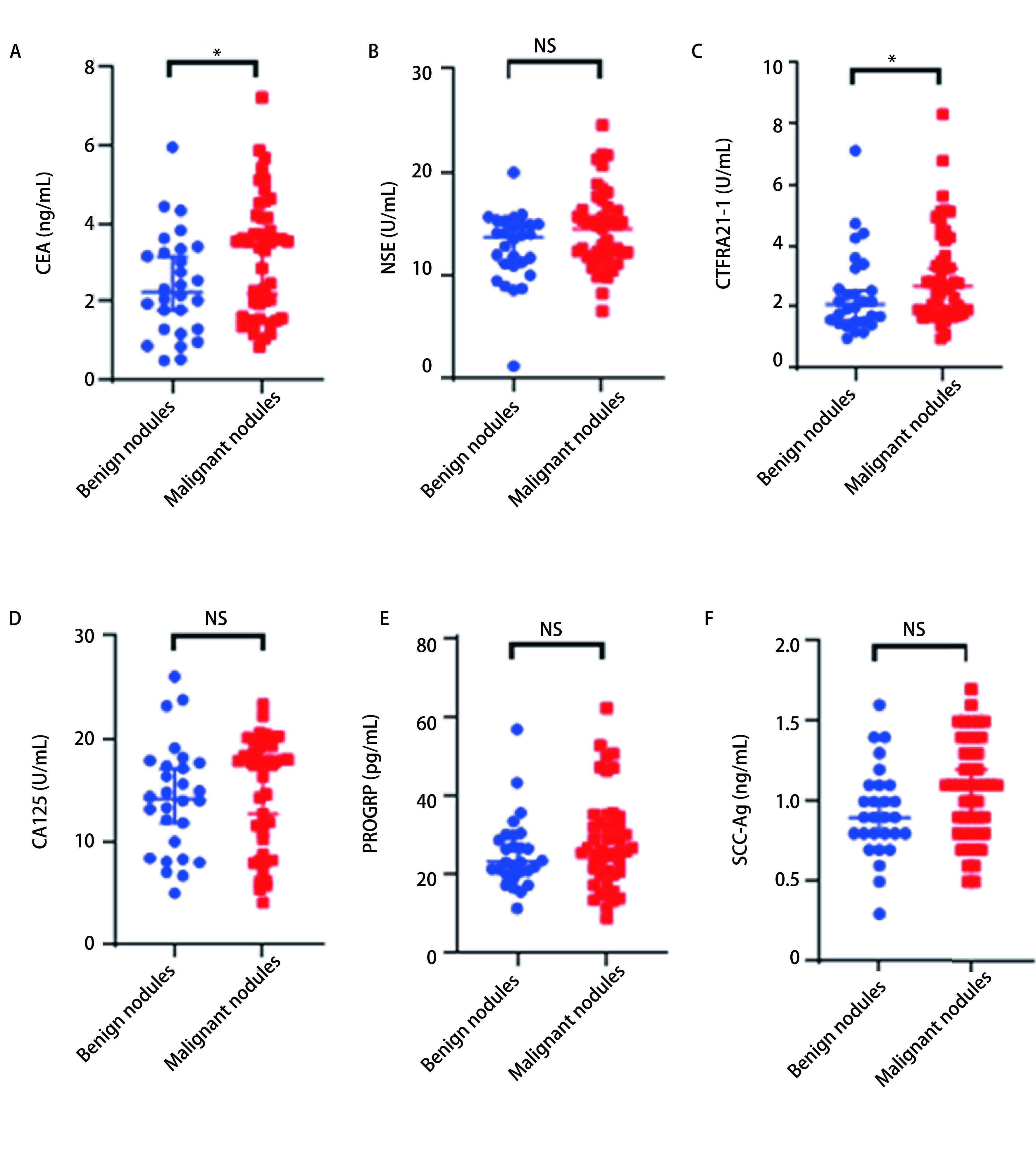
良性结节组和恶性结节组中肿瘤标志物表达水平的比较 Comparison of tumor markers expression levels of patients with benign or malignant solitary pulmonary nodules. *: *P* < 0.05.

**表 3:** 肿瘤标志物在良性结节组和恶性结节组表达水平 Tumor markers expression level of patients with benign or malignant solitary pulmonary nodules

Tumor marker	CEA（ng/mL）	NSE（ng/mL）	CYFRA21-1(ng/mL)	CA125(U/mL)	PROGRP(pg/mL)	SCC-Ag(ng/mL)
Benign solitary pulmonary nodules (*n*=34)	2.25(1.31, 3.31)	13.72(10.96, 14.97)	2.09(1.52, 2.93)	15.27(8.08, 18.13)	22.90(20.50, 29.10)	0.90(0.8, 1.10)
Malignant solitary pulmonary nodules (*n*=47)	3.39(1.62，4.15)	14.53(11.85, 16.33)	2.68(1.80, 4.16)	17.59(10.29, 19.31)	25.70(19.80, 32.90)	1.10(0.8, 1.30)
*Z* (*t*)	-2.112	-1.354	-2.373	-2.105	-0.880	-1.521
*P*	0.038	0.176	0.047	0.232	0.379	0.128
CEA: carcinoembryonic antigen; NSE: neuron-specific enolase; CYFRA21-1：cytokeratin 19 fragment 21-1; CA125: carbohydrate antigen 125; PROGRP: progastrin releasing peptide; SCC-Ag: squamous cell carcinoma antigen.						

### 2.4 细胞因子和肿瘤标志物的单因素及多因素分析结果

由二元*Logistic*单因素分析可知（表 4），CEA、IL-6及IL-8有助于恶性结节的诊断，为恶性SPN的危险性因素，NSE、CYRFA、CA125、PROGRP、SCC、IL-2R、TNF-α对恶性结节的诊断均无显著统计学差异，因此不纳入多因素回归分析。

**表 4:** 恶性结节中细胞因子和肿瘤标志物血清水平的单因素分析结果 Result of univariate analysis of cytokines and tumor markers in patients with malignant solitary pulmonary nodules

Cytokines and tumor markers	B	Walds	OR	95%CI	*P*
CEA	0.372	4.108	1.450	1.012-2.077	0.043
IL-6	0.268	5.359	1.307	1.042-1.640	0.021
IL-8	0.136	7.904	1.145	1.042-1.259	0.005

基于单因素分析的结果及各因素之间可能存在的相互作用，将CEA、IL-6和IL-8纳入多因素回归分析中，得出结果如表 5所示，CEA、IL-6和IL-8为预测恶性结节的独立危险因子，它们的OR值分别为1.620（95%CI: 1.043-2.517, *P* < 0.05）、1.317（95%CI: 1.028-1.688, *P*=0.030）和1.122（95%CI: 1.027-1.226, *P*=0.011）。

**表 5:** 恶性结节中细胞因子和肿瘤标志物血清水平的多因素分析结果 Result of multivariate analysis of cytokines and tumor markers in patients with malignant solitary pulmonary nodules

Cytokines and tumor markers	B	Walds	OR	95%CI	*P*
CEA	0.482	4.604	1.620	1.043-2.517	0.032
IL-6	0.275	4.730	1.317	1.028-1.688	0.030
IL-8	0.115	6.457	1.122	1.027-1.226	0.011

### 2.5 细胞因子及其与肿瘤标志物联合检测的ROC分析

我们通过二元*Logistic*回归分析构造联合因子CEA+IL-6、CEA+IL-8和CEA+IL-6+IL-8，用联合因子的概率值代表指标联合检测水平，分别用3个单项指标及其联合构造的指标绘制ROC曲线，计算AUC，并检验AUC的差异是否具有统计显著性，取Youden指数最大值时的阈值作为最佳诊断临界值，计算对应的灵敏度、特异度、阳性预测值、阴性预测值和准确率。

单项指标CEA、IL-6、IL-8及联合检测分组（CEA+IL-6、CEA+IL-6和CEA+IL-6+IL-8）的ROC曲线分析结果见图 3及表 6。CEA、IL-6、IL-8的最佳诊断临界值分别是3.455 ng/mL、4.160 pg/mL和9.710 pg/mL。ROC曲线分析结果显示，指标联合检测的AUC均大于单项指标，依次是CEA+IL-6+IL-8 > CEA+IL-8 > CEA+IL-6 > IL-8 > IL-6 > CEA。

**图 3:**
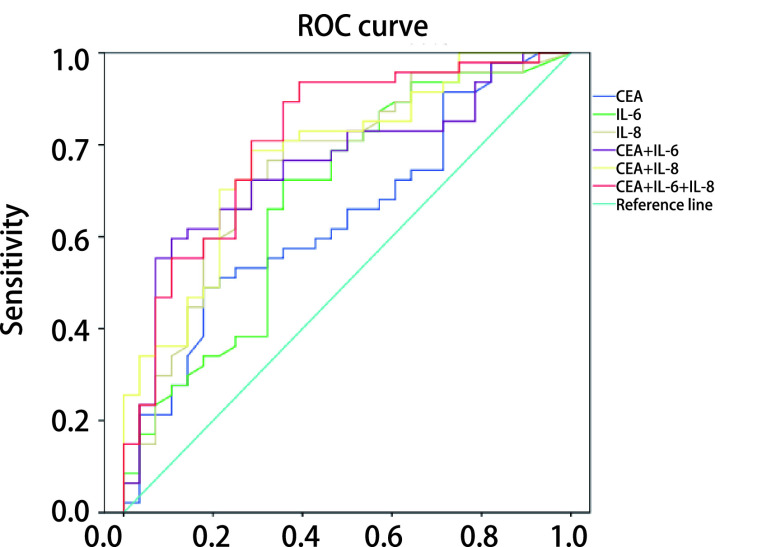
不同指标对恶性孤立性肺结节诊断的ROC曲线 ROC curve of different index in malignant solitary pulmonary nodules

**表 6:** 不同指标对恶性孤立性肺结节诊断的ROC曲线分析 ROC curve analysis of different index in malignant solitary pulmonary nodules

Index	AUC	*P*	95%CI	Cutoff	Youden index	Sensitivity	Specificity	Positive prediction value	Negative prediction value	Accuracy
CEA	0.642	0.041	0.514-0.770	3.455	0.311	48.9%	82.1%	82.1%	48.9%	61.3%
IL-6	0.684	0.008	0.555-0.813	4.160	0.366	72.3%	64.3%	77.3%	58.1%	69.3%
IL-8	0.749	0.000	0.631-0.866	9.710	0.451	80.9%	64.3%	79.2%	62.1%	74.7%
CEA+IL-6	0.754	0.000	0.641-0.867	-	0.489	59.6%	89.3%	90.3%	56.8%	70.7%
CEA+IL-8	0.776	0.000	0.668-0.884	-	0.502	78.7%	71.4%	82.2%	66.7%	76.0%
CEA+IL-6+IL-8	0.812	0.000	0.708-0.915	-	0.543	93.6%	60.7%	80.0%	85.0%	81.3%
ROC: receiver operating characteristic curve.										

## 3 讨论

肺癌是最常见的癌症，也是癌症相关死亡的主要原因。随着环境问题的加剧，肺癌的负担日益增加。尽管肺癌的诊疗不断取得进展，但其预后和生存期仍无明显改善，并且随着分期的增加，生存率显著下降。因此，肺癌的早期筛查与诊断具有重要临床意义。随着健康体检筛查的普及和胸部CT的广泛应用，SPN的检出率显著增加，部分SPN是早期肺癌的表现，其良恶性的鉴别是目前临床重点关注的问题，尽早识别SPN中的恶性结节，并及时治疗，可以显著改善肺癌患者的预后和生存率。

一些患者的临床特征被认为是肺部恶性肿瘤的危险因素，比如年龄的增长、吸烟史、肿瘤家族病史、职业暴露、肺纤维化和慢性阻塞性肺疾病等^[7]^。已有研究^[8]^表明，肺癌发病率的风险随着年龄的增长而显著增加，在≥65岁的SPN患者中，恶性率超过85%，是年轻组的2.25倍，提示年龄是判断SPN为恶性的独立影响因素。本研究纳入的恶性SPN患者中， < 65岁的人群中恶性SPN占38.3%，≥65岁的人群中恶性SPN占61.7%，这与上述研究结果一致。另外，吸烟是肺癌发生的主要危险因素，在吸烟者中恶性肺结节的生长更快，容易促进病情向晚期进展。在初次筛查时，50岁以上的吸烟患者中几乎都会发现肺部结节，并且有10%的筛查对象会在1年内出现新的结节^[9]^。本研究中良性和恶性SPN病例在年龄、性别、吸烟史和家族史中分布无显著差异，但这并不意味着这些临床特征与恶性SPN无相关性，可能与样本量较小有关。在将来的研究中，应该扩大样本量、丰富研究因素，进行多中心研究，进一步探讨SPN的流行病学因素。

另外一些影像学特征有助于SPN患者的危险分层，包括结节大小、密度、位置、边缘及内部特征和体积倍增时间等。在美国国家癌症研究所的肺部筛查研究中， > 20 mm的结节的肺癌诊断率最高，然后依次是11 mm-19 mm和≤10 mm的结节，分别为34.5%、21.3%和 < 5%^[10]^。SPN直径越大恶性概率的可能性越高，本研究47例恶性SPN中20 mm-30 mm、11 mm-19 mm和≤10 mm所占的比例依次为51.1%、40.4%和8.5%，与既往研究一致。本研究中SPN在右肺上叶发生的比例最高（30.7%），其中恶性SPN位于右肺上叶的比例为40.4%，这与大多数的研究是一致的。Takahashi等^[10]^对360例有病理结果的SPN患者进行分析，肺结节多位于右肺上叶（37.5%），大多数≤20 mm的恶性SPN位于右肺上叶（41.4%）。研究^[10]^发现在3种不同密度的结节中，部分实性结节的恶性概率最高，然后依次是实性结节、磨玻璃样结节。本研究81例SPN患者中，部分实性结节、实性结节和磨玻璃样结节中恶性结节的概率依次为60.0%、62.7%和40%，与上述研究结果不一致，但符合大多是恶性结节是实性结节的流行病学规律。可继续随访收集更多部分实性结节和磨玻璃样结节的病例纳入研究，展开深入分析。

CEA是一种参与细胞黏附的多糖蛋白复合物，在健康成年人的血液中它通常不存在或含量甚微，被认为与肿瘤的不良预后相关^[11]^。CEA血清学水平与肺癌的病理分期关系密切。Grunnet等^[11]^发现肺癌患者血清中CEA较良性病变患者升高更明显（*P* < 0.05），但早期变化不大。我们的研究结果中，CEA是恶性SPN的独立预测因素，其特异度是几项单独指标中最高的，但灵敏度仅为48.9%，说明CEA在早期肺癌中的作用有限。

为了进一步解决CEA在早期肺癌诊断中的局限性，与其他血清生物标志物进行联合检验成为目前研究的热点。细胞因子作为连接肿瘤细胞、炎性微环境和宿主免疫系统的纽带，与肺癌的发生、发展紧密相连。既往研究^[12]^发现IL-6可通过诱导血管扩张共济失调突变基因（ataxia-telangiectasia mutated kinase, ATM）磷酸化，增加基质金属蛋白酶（matrix metalloproteinase, MMP）表达，促进肺癌的上皮间充质转化，而IL-6/STAT3信号传导通过诱导细胞增殖调节剂细胞周期蛋白D1促进了肺癌细胞的生长^[13]^。IL-8促进肺癌的机制尚不明确，有研究^[14]^认为IL-8通过GLUT3和GFAT诱导的O-GlcNAc修饰可调节结肠和肺癌细胞中的癌干细胞样特性，这些机制研究均说明IL-6及IL-8在肺癌的发生和发展中起到重要作用。Holmer等^[15]^研究发现，与健康对照组相比，非小细胞肺癌（non-small cell lung cancer, NSCLC）患者血清中IL-8、IL-6、TNF-α和血管内皮生长因子（vascular endothelial growth factor, VEGF）水平明显升高。Pine等^[16]^研究证明，高水平的IL-6和IL-8患者肺癌发病率分别增加了3.29倍和2.06倍，表明IL-6和IL-8是肺癌的危险因素，这与我们的结果相符。本研究中，IL-6、IL-8在恶性结节组中的表达水平显著高于良性结节组（*P* < 0.05），和既往研究一致。但在肺腺癌和肺鳞癌两个NSCLC病理分型亚组间，IL-6的表达水平无差异，肺鳞癌组IL-8的表达水平高于肺腺癌组，这说明IL-6和IL-8在恶性结节患者中的表达水平升高，IL-8可能与组织学亚型肺鳞癌具有一定的相关性，但是本研究中鳞癌样本量偏少，可能存在偏倚，需扩大样本量进一步研究。本研究单因素及多因素分析发现IL-6和IL-8的升高与恶性SPN存在密切相关。本研究提示，外周血细胞因子可能是早期肺癌的血清标志物，反映癌症的存在和机体特异性的高免疫反应。

本研究对有独立预测意义的CEA、IL-6、IL-8这3项血清指标及其不同联合检测组合进行综合分析评价。将CEA分别与IL-6、IL-8组合联合检测，有3种不同的组合方式（CEA+IL-6、CEA+IL-8和CEA+IL-6+IL-8），其中CEA+IL-6+IL-8的灵敏度最高，并且其诊断准确率在单项检测和联合检测中最高，综合评价分析认为3项指标联合检测的诊断价值高于CEA、IL-6、IL-8单独诊断效能以及两两诊断效能，说明CEA+IL-6+IL-8联合检测有望辅助诊断SNP的性质，对肺癌的诊断有一定的价值。

综上所述，肿瘤标志物和细胞因子联合检测对恶性SNP的早期诊断较单项更具有临床意义，提高了对肺部恶性肿瘤的诊断效能，可以作为辅助检查手段，为临床提供更多的参考信息和诊断证据。
